# Silver Nanoparticles Exhibit the Dose-Dependent Anti-Proliferative Effect against Human Squamous Carcinoma Cells Attenuated in the Presence of Berberine

**DOI:** 10.3390/molecules21030365

**Published:** 2016-03-17

**Authors:** Arkadiusz Dziedzic, Robert Kubina, Rafał J. Bułdak, Magda Skonieczna, Krzysztof Cholewa

**Affiliations:** 1 Department of Conservative Dentistry with Endodontics, School of Medicine with the Division of Dentistry, Medical University of Silesia in Katowice, Pl. Akademicki 17, Bytom, 41-902, Poland; 2 Department and Institute of Pathology, School of Pharmacy and Division of Laboratory Medicine in Sosnowiec, Medical University of Silesia in Katowice, ul. Ostrogórska 30, Sosnowiec 41-200, Poland; rkubina@sum.edu.pl; 3 Department of Physiology, School of Medicine with the Division of Dentistry, Medical University of Silesia in Katowice, ul. H. Jordana 19, Zabrze 41-808, Poland; Erbuldak@sum.edu.pl; 4 Department of Automatic Control—Biosystems Groups, Silesian University of Technology, Gliwice 44-100, Poland; magdalena.skonieczna@polsl.pl; 5 Department of Biochemistry, School of Pharmacy with the Division of Laboratory Medicine, Medical University of Silesia in Katowice, Sosnowiec 41-200, Poland; kcholewa@sum.edu.pl

**Keywords:** silver nanoparticles, berberine, oral carcinoma, MTT assay, SCC-25, cell cycle arrest, bax/bcl-2 gene expression, RT-QPCR

## Abstract

The biological activity of nanosize silver particles towards oral epithelium-derived carcinoma seems to be still underinvestigated. We evaluated the influence of low doses of nanosize scale silver particles on the proliferation and viability of malignant oral epithelial keratinocytes *in vitro*, alone and in conjunction with the plant alkaloid berberine. Cells of human tongue squamous carcinoma SCC-25 (ATCC CRL-1628), cultivated with the mixture of Dulbecco's modified Eagle’s medium, were exposed to silver nanoparticles alone (AgNPs, concentrations from 0.31 to 10 μg/mL) and to a combination of AgNPs with berberine chloride (BER, 1/2 IC_50_ concentration) during 24 h and 48 h. The cytotoxic activity of AgNPs with diameters of 10 nm ± 4 nm was measured by 3-(4,5-dimethyl-2-thiazyl)-2,5-diphenyl-2*H*-tetrazolium bromide (MTT) assay. Cell cycle analysis was performed by treating cells with propidium iodide followed by flow-activated cell sorting. RT-QPCR reaction was used to assess expression of anti-apoptotic proteins Bcl-2 and pro-apoptotic protein Bcl-2-associated X protein Bax genes expression. Monodisperse silver nanoparticles at a concentration of 10 μg/mL arrested SCC-25 cells cycle after 48 h at the G0/G1 phase in a dose- and time-dependent manner through disruption G0/G1 checkpoint, with increase of Bax/Bcl-2 ratio gene expression. AgNPs exhibit cytotoxic effects on SCC-25 malignant oral epithelial keratinocytes, which is diminished when combined with BER. The AgNPs concentration required to inhibit the growth of carcinoma cells by 50% (IC_50_) after 48 h was estimated at 5.19 μg/mL. AgNPs combined with BER increased the expression of Bcl-2 while decreasing the ratio of Bax/Bcl-2 in SCC-25 cells. Silver particles at low doses therefore reduce the proliferation and viability of oral squamous cell carcinoma cells. SCC-25 cells are susceptible to damage from AgNPs-induced stress, which can be regulated by the natural alkaloid berberine, suggesting that nanoparticles may be potentially used in a chemoprevention/chemotherapy by augmentation of action of standard anti-cancer drugs.

## 1. Introduction

Oral squamous cell carcinoma (OSCC), defined broadly also as head & neck squamous cell carcinoma (HNSCC) comprises a group of epithelial cancers originated from e.g., the oral or nasal cavity, lips, paranasal sinuses, and also pharynx or larynx [1]. HNSCC is responsible for about 6% of all malignant diseases diagnosed, and is thus currently the eighth leading cause of cancer death worldwide, with an incidence of more than 600,000 new cases per year [2,3]. About 40% of intraoral squamous cell carcinomas begin on the floor of the mouth or on the lateral and ventral surfaces of the tongue. A vast majority of patients present with advanced tumors or regional lymph node metastases resulting in a poor prognosis [4].

Despite early diagnostic efforts, advanced surgery, radiotherapy, chemotherapy or various combination of them, the survival rate of patients with oral cancer, cancer incidence and mortality have not improved significantly [5]. Moreover, a substantial number of patients treated due to OSCC may develop a second cancer lesion within a few years. Due to the distinct localization of these tumors in regions with anatomic structures important to e.g., breathing, mastication, swallowing, invasive treatment regimens frequently lead to severe functional impairments and unfavorable cosmetic outcomes [6]. Additionally to that, radiotherapy may have long-term effects on surrounding healthy structures such as skin, brain, or salivary glands. On the other hands, chemotherapy applied systemically may result in severe adverse effects and affect blood cell production leading to the impairment of homeostasis: anemia, neutropenia, and/or thrombopenia [7].

A variable types of biologically active nanoparticles have been used during the XIXth century due to their health benefits, however validated studies of their effect on malignant tumor cells have only begun within the last few decades. The unique properties of silver nanoparticles (AgNPs) make them suitable for numerous technologies, including biomedical, materials, and antimicrobial applications, as well as for use in nanotoxicology studies [8,9]. Nowadays, the most common application of AgNPs is their use as an antimicrobial measure and in antiseptic wound dressings. Medical devices such as stents and catheters may gradually release a low level of silver ions to maintain an efficient protection against bacteria [10,11,12]. In all studies to date, silver nanoparticle toxicity towards vital tissues is much less than the equivalent mass loading of silver salts and chemical compounds [13].

Chemotherapy of oral squamous cell carcinoma is not used routinely as a primary anti-cancer treatment but it can be recommended as an adjuvant method in combination with radiation therapy in individuals with advanced malignant tumor progression. Nowadays, standard chemotherapeutic agents are used mainly as a supportive treatment of OSCC, including doxorubicin, daunorubicin, bleomycin, and cisplatin [14]. However, they are known to induce severe side effects such as: myelosuppression and anemia [15]. For that reason, it is important to find alternative therapies or drugs to overcome these drawbacks.

The cytotoxicity of AgNPs has been investigated in various cell models [16,17]. These *in vitro* studies have revealed that AgNPs are able to interfere with cellular functions and cause toxic effects, including DNA damage and apoptosis, also in normal cells [18,19]. The available research data focus mainly on the diagnostic value of nanoparticles in oncology, and research on the anticancer effects of silver nanomolecules is lacking. To our knowledge, silver nanoparticles at relatively low concentrations, not affecting normal cells, have not yet been investigated in the context of on their anti-tumor potential towards oral cancers. The anti-proliferative effect of the alkaloid berberine (BER) on squamous carcinoma cells was elucidated in *in vitro* studies [20,21,22], however, there is no research investigating the combined biological, cellular effect of AgNPs in low concentrations in combination with this compound from a medicinal plant—berberine.

There are recent reports on the anti-proliferative effect of silver nanoparticles on human breast cancer cells MCF-7 [23,24], human glioblastoma cells U251 [25] and chronic myeloid leukemia cells under *in vitro* conditions [26]. Here, firstly we assessed the *in vitro* biological behavior of low concentrations of silver-based nanoparticles on the OSCC cell line SCC-25 alone. The second aim of this study was to investigate the possible interactions of AgNPs and the natural alkaloid berberine, with regard to their cytotoxicity and influence on malignant oral epithelial keratinocyte viability. The clinical relevance of this article lies in its focus on the biological effects of silver nanoparticles alone and in conjunction with BER, and their potential clinical use as an adjuvant for chemotherapy of squamous cell carcinoma the tongue and mouth or oropharynx. The protocol with the use of AgNPs + BER would provide a new way for their practical application as a novel regulatory method for chemotherapy delivery.

## 2. Results and Discussion

The experiments were aimed at determining whether the addition of bio-active silver particles of selected nanosize scale may inhibit the proliferation and viability of oral cancer cells, as recent reports have confirmed the role of nanoparticle-induced cellular stress on selected tumor cells [23,24,25,26]. The effect of the addition of the AgNPs on the oral squamous cancer cell line, SCC-25 was investigated *in vitro* in a micro-culture system using various incubation concentrations. Cytotoxicity of AgNPs was determined as the percentage of viable SCC-25 carcinoma cells at different concentrations of AgNPs with regards to the unexposed cells. Additionally, the half maximal Inhibitory Concentration (IC_50_) was defined as the AgNP concentration value which is required to inhibit the viability of SCC-25 cells in culture by 50% compared to the untreated cells. IC values were extrapolated from cell viability-AgNPs concentration curves. To find out the minimum AgNPs concentration required to cause effects of 50% growth inhibition in SCC-25 cells after 24 h and 48 h, a log_viability_–log_dose_ curve was plotted.

### 2.1. Effect of Low Doses of AgNPs on SCC-25 Cell Line Viability and Mitochondial Function

**Figure 1:**
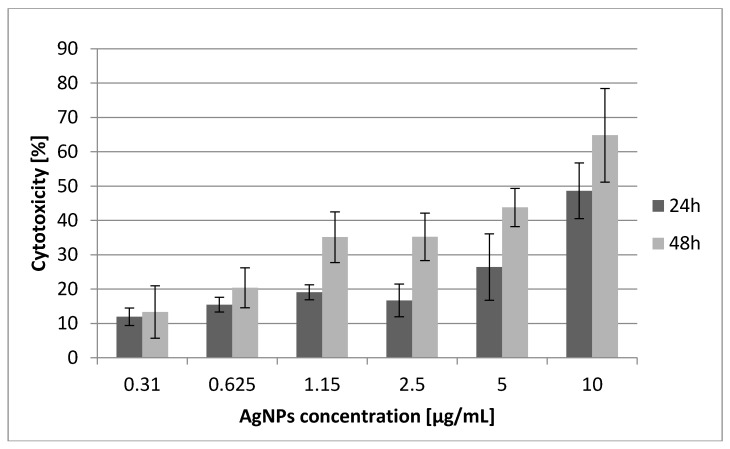
Cytotoxic effects of silver nanoparticles (10 nm diameter, concentrations 0.31 μg/mL–10 μg/mL) on SCC-25 cancer cells. The percentage of cell death measured by MTT cytotoxicity assay. MTT values represent mean ± SD of three independent cytotoxicity experiments performed in quadruplicate (*n* = 12). The lower concentration of AgNPs (e.g., 0.625 μg/mL) after 48 h produced the same killing effect on SCC-25 cells (20%) as 3 μg/mL AgNP concentration after 24 h. Mean cytotoxicity between different AgNPs concentrations alone were highly significant above the concentration of 2.5 μg/mL (*p* < 0.01, ANOVA Friedman ANOVA test, Wilcoxon test).

As shown in Figure 1, AgNPs alone (10 nm particle size) at concentrations of 0.31 μg/mL–10 μg/mL induced cytotoxic effects on SCC-25 carcinoma cells in a dose-dependent manner and displayed a time-dependent cytotoxic effect during 24 h and 48 h of experiment. However, AgNP concentrations within the range 1.25 μg/mL–2.5 μg/mL did not alter the SCC-25 cells’ viability and indirect proliferation during 24 h and 48 h of exposure, reflected by a slight absorbance increase for 24 h incubation time (Figure 1). The minimum AgNPs concentrations required to cause 20, 25, 40 and 50% cell growth inhibition after 48 h were 0.56, 0.81, 2.47 and 5.19 μg/mL respectively, while the IC_20_, IC_25_, IC_40_ and IC_50_ values for 24 h of incubation time were: 1.25, 2.21, 12.14 and 37.87 μg/mL. The last values (12.14 and 37.87) were estimated mathematically using extrapolation from the obtained data.

The dose of AgNPs required to inhibit growth of 50% of SCC-25 cells (IC_50_) decreased with a longer incubation time of 48 h. Additionally, during the experiment the IC_50_ value for berberine chloride (BER) was established as 25 μg/mL. The results of our cytotoxicity studies using the MTT assay reveal that this cell line is susceptible to ultra-low size silver nanoparticles after 48 h of exposure, with an IC_50_ value (5.19 μg/mL) not exceeding the tested AgNP concentrations. On the other hand, SCC-25 cells seem to be relatively resistant to AgNPs up to 24 h of exposure, with an IC_50_ value significantly exceeding the tested AgNP concentration of 10 μg/mL (37.87 μg/mL). Differences in MTT absorbance mean values between 24 h and 48 h of incubation time samples, considering the same AgNP concentrations, were statistically significant for AgNP concentrations above 1.15 μg/mL (Wilcoxon test, *p* < 0.05).

**Figure 2:**
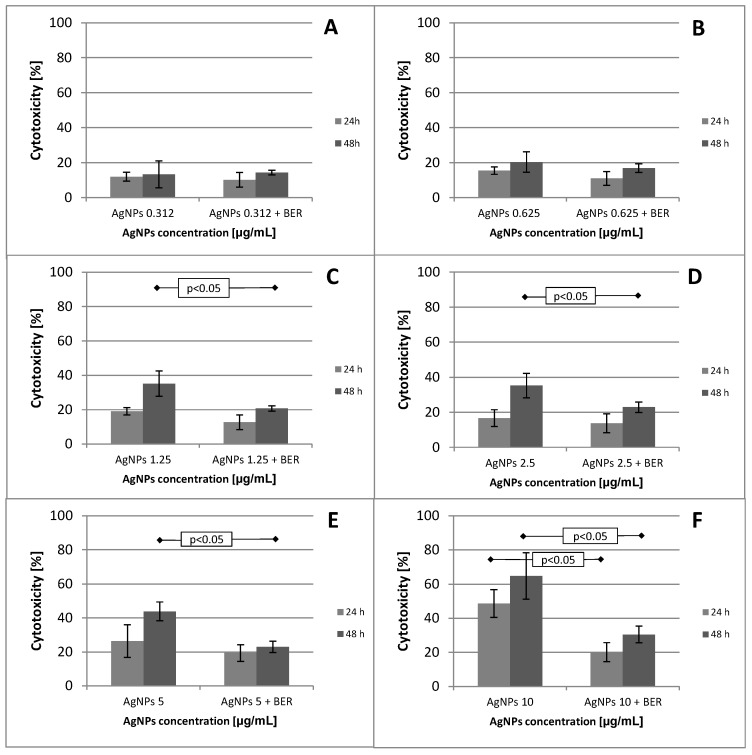
MTT assay results after 24 and 48 h of cells culture incubation representing mean values for different concentrations of AgNPs alone and in combination with a fixed 1/2 IC_50_ (12.5 μg/mL) concentration of BER. AgNPs with BER inhibited significantly less the growth of SCC-25 in a dose-dependent manner which clearly demonstrates an attenuating effect of BER addition, particularly at 5 and 10 μg/mL concentrations (**A**–**F**). Differences of mean cytotoxicity values between different AgNPs concentrations alone *vs*. AgNPs/BER were significant (*p* < 0.05 and *p* < 0.01, ANOVA Friedman ANOVA test).

**Figure 3:**
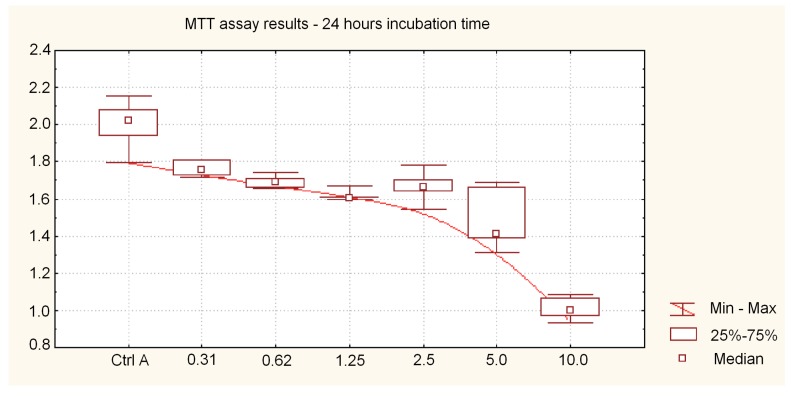
Assessment of cell viability after treatment with various nanosilver particle concentrations during 24 h. Cell viability compared to the control (untreated cells) was plotted against the absorbance at 570 nm. MTT assay results after 24 h of incubation representing absorbance values (median, min-max) for 0.31 μg/mL to 10 μg/mL AgNP concentrations. AgNPs inhibited the cells’ growth in a dose-dependent manner. Differences of mean absorbance values between different AgNPs concentrations were highly significant (*p* < 0.01, ANOVA Friedman ANOVA test).

**Figure 4:**
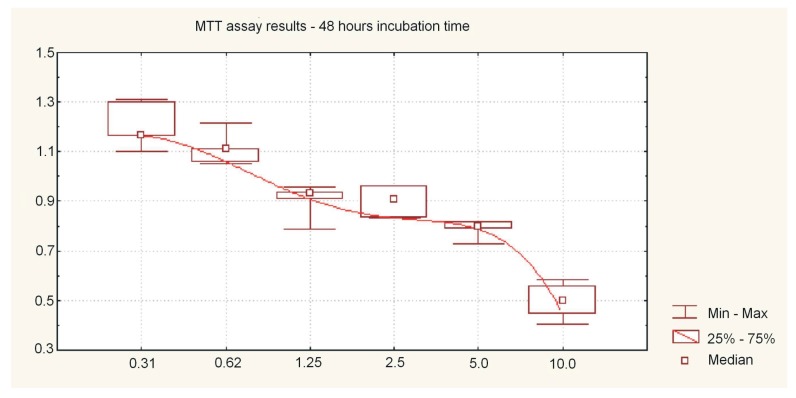
MTT assay results after 48 h of cell culture incubation representing absorbance values (median, min-max) for different dispersed Ag-NP concentrations (0.31 μg/mL to 10 μg/mL). Like after 24 h incubation time, AgNPs also inhibited the growth in a dose-dependent manner, with a significant variation of MTT absorbance values compared to the 24 h protocol. A “flattened” viability curve between the concentrations of 1.5 and 5 μg/mL of AgNP could be an effect of the experimental conditions. Differences of mean absorbance values between different AgNPs concentrations were highly significant (*p* < 0.01, ANOVA Friedman ANOVA test).

Cell viability of SCC-25 cells treated with 10 nm AgNPs and AgNPs + BER is shown in Figure 1 and Figure 2, respectively. The hostile influence of AgNPs on SCC-25 cells was found to be considerably reduced in the presence of 12.5 μg/mL BER (1/2 IC_50_) for AgNP concentrations above 1.25 μg/mL. The cytotoxicity of the combined AgNPs + BER mixture decreased 2-fold as compared with AgNPs 10 μg/mL action alone. This “diminishing phenomenon” can be caused by physical/chemical properties, particularly negatively surface charged nanoparticles which hypothetically produce a coating layer on the berberine molecules. This explains the lesser potential toxicity of AgNPs in the presence of BER. However, the attenuating effect if not significant with regards to the lowest AgNPs concentrations, hypothetically due to lesser amount of nanoparticles able to cover the BER compound. The cytotoxicity of 0.31–10 μg/mL AgNPs after 48 h incubation was 13.34% ± 7.66%–64.80% ± 13.65% cell death (*p* < 0.05), *vs.* 11.95% ± 2.54%–48.64% ± 8.08% (*p* < 0.05) after 24 h. As shown no significant effects were noticed till 24 h of incubation considering the AgNP concentrations of up to 2.5 μg/mL. After 48 h the cytotoxic effects became obvious. Differences of mean absorbance values within the same incubation time (24 h or 48 h), between different AgNPs concentrations were highly significant (*p* < 0.01, ANOVA Friedman ANOVA test). The overall trends, including non-linear decreasing MTT absorbance value (median, min-max) for different dispersed AgNP concentrations, 24 h and 48 h incubation time are presented in Figure 2, Figure 3 and Figure 4.

Here, we report that the tested nanoparticles at concentrations up to 2.5–5 μg/mL exhibit relatively low cytotoxic activity against oral cancer cells. As shown in Figure 3 and Figure 4, after exposure to 1.15–2.5 μg/mL of 10 nm AgNPs for 24 h, the cell viability actually increased slightly. The absorbance value significantly decreased and cytotoxicity increased significantly for AgNP concentrations above 5 μg/mL (*p* < 0.05). These effects were determined by measuring the dose required to inhibit growth of 50% of cells (IC_50_) which exhibited a value of 5.19 μg/mL after 48 h of incubation.

### 2.2. Effect of Low Doses of AgNPs on SCC-25 Cell Cycle Phase Distribution

**Figure 5:**
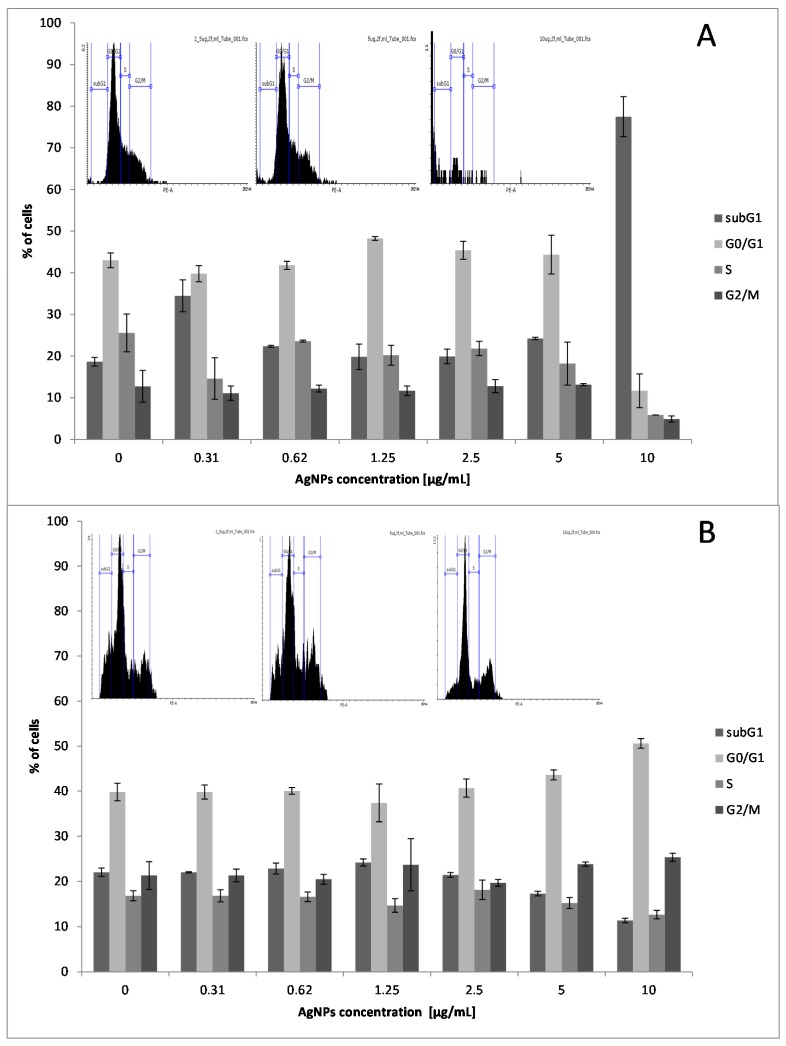
Representative flow cytometric histograms and bar graphs showing the cell cycle distribution when the SCC-25 cells were treated with AgNPs at 0.31, 0.62, 2.5, 5, 10 μg/mL for 24 h (**A**) and 48 h (**B**). Cells were stained with propidium iodide and subjected to flow cytometric analysis that collected 10,000 events. Alterations in the percentage of SCC-25 cells in subG1, G0/G1, S, and G2/M phases of cell cycle are presented as the mean ± SD of three independent experiments. The results show that AgNPs (10 µg/mL) has a significant effect on cell cycle arrest, contributing to their anticancer features. AgNPs treatment of SCC-25 with 10 µg/mL concentration for 24 h and 48 h resulted in a cell cycle (checkpoint) arrest within the subG1 and G0/G1 phase, respectively (*p* < 0.01 and *p* < 0.05, independent experiments).

**Figure 6:**
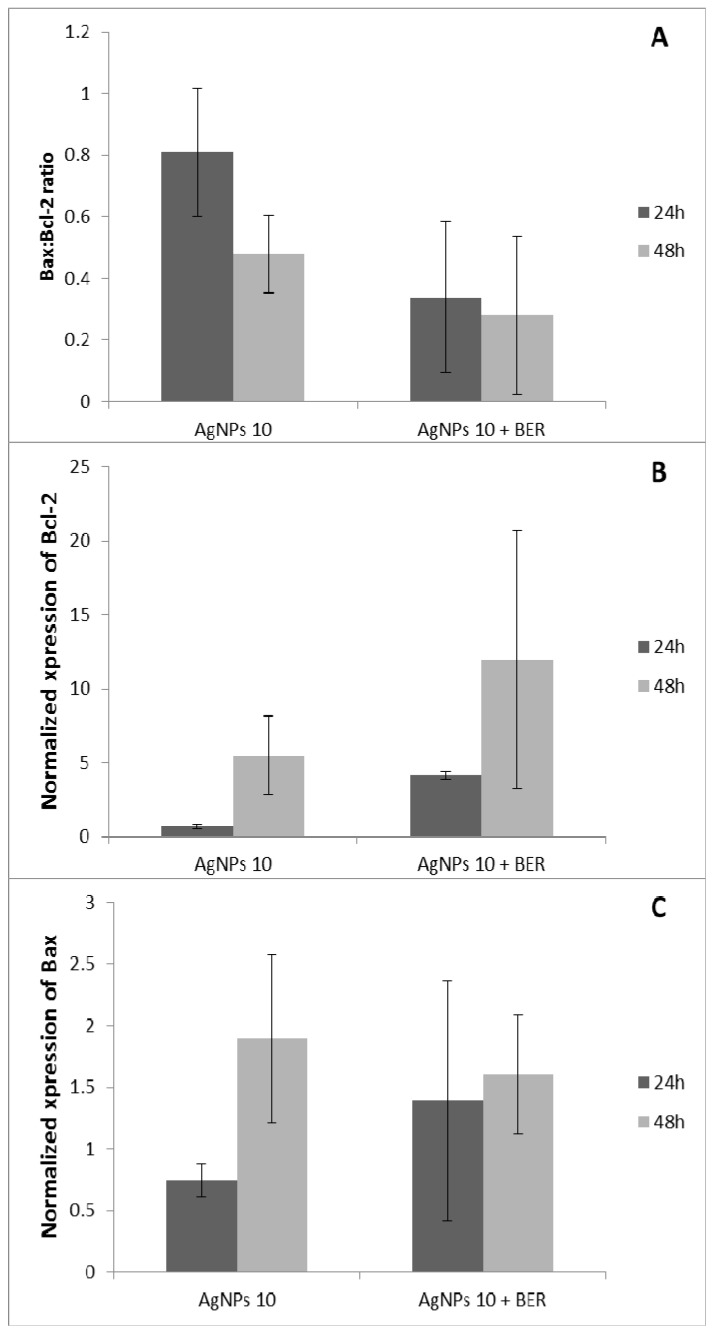
Effect of AgNPs and combined action of AgNPs + BER on the expression levels of apoptosis-related genes Bax/Bcl-2 in SCC25 cells. The expression levels of Bax, Bcl-2 in SCC-25 cells were determined by RT-QPCR assay. Specificity of the reaction of amplification of all fragments of studied genes was assessed by means of polyacrylamide gel electrophoresis. Each sample was examined in two repetitions. (**A**) Representative bar graphs show the expression of relative level of Bax/Bcl-2 ratio, when the cells were treated with AgNPs at 10 μg/mL and combination of AgNPs/BER for 24 and 48 h (*p* < 0.05, ANOVA, AgNPs *vs.* AgNPs + BER); (**B**) Representative bar graphs show the expression of level of Bcl-2 gene, when the cells were treated with AgNPs at 10 μg/mL and combination of AgNPs/BER for 24 and 48 h (*p* < 0.05, ANOVA, AgNPs *vs.* AgNPs + BER); (**C**) Representative bar graphs show the expression of level of Bax gene, when the cells were treated with AgNPs at 10 μg/mL and combination of AgNPs/BER for 24 and 48 h.

Because previous reports found that AgNPs modulate the expression of regulatory proteins involved in cell cycle progression [27,28], their effect on SCC-25 cell cycle status was examined. In order to explore further the anti-proliferative effect of AgNPs on SCC-25 cells, the effect of AgNPs on the cellular cycle distribution was quantified using flow cytometric analysis. Due to the fact that the MTT assay revealed an attenuating effect of BER addition on SCC-25 exposed to AgNPs, flow cytometry analysis was only applied for AgNPs alone. The investigated cells were exposed to 0.31, 0.62, 2.5, 5, and 10 μg/mL AgNPs for 24 h. The cell cycle distribution within 24 h showed no significant change form 0.31 up to 5 μg/mL, whereas there was a significant increase in cell numbers in the subG1 phase when treated with 10 μg/mL for 24 h (*p* < 0.01, Figure 5A). Treatment with 10 µg/mL for 24 h and 48 h resulted in a significant accumulation of cells in the subG1 and G0/G1 phase, respectively, for SCC-25 cell line (*p* < 0.01 and *p* < 0.05, Figure 5A,B). This effect can be associated with 10 µg/mL AgNPs-induced upregulation of cell-cycle regulators associated with Bax gene expression following 48 h of experiment (Figure 6A,C). Cell numbers in the G0/G1/S phases and G2/M phase were markedly decreased when exposed to 10 μg/mL for 24 h and 48 h, respectively, compared to the control. This finding suggests an anti-viability activity of relatively low concentrations of AgNPs in malignant cells and is consistent with a previous reports [28] regarding *in vitro* squamous cell carcinoma cells studies.

### 2.3. Bcl-2 and Bax Gene Expression by Analyzed by RT-QPCR

The regulatory mechanisms of cell apoptosis include regulation of Bax, and Bcl-2 gene expression [29,30], activation of death receptors, mitochondrial responses and caspases. The products of Bcl-2 and Bax genes and the relative levels of the available dimerization partners shift the balance of cell fate in favor of either viability or cell death. We demonstrated a relatively inverse relationship between the Bcl-2 and Bax gene expression. A remarkable effect of the combination of AgNPs 10 μg/mL and BER on the regulation of Bax/Bcl-2 genes expression was observed, with appr. 40% reduction of Bax/Bcl-2 index (*p* < 0.01, ANOVA) (Figure 6A). Pro-apoptotic Bax gene expression was significantly increased after treatment with AgNPs alone at 10 μg/mL considering 24 h *vs.* 48 h (*p* < 0.05, Figure 6C). As our results showed that Bax and Bcl-2 gene expression are regulated differently by AgNPs alone and in combination with the alkaloid berberine chloride, which suggests that a balance in the expression of these genes might be responsible for the control of the apoptosis.

On the other hand, a combination of AgNPs and BER induced markedly Bcl-2 gene expression and activation of anti-apoptotic pathway, which reveals an obvious decrease of AgNP pro-apoptotic action in the presence of berberine (*p* < 0.05, Figure 6B). This indicates that the lesser activity of AgNPs + BER seems to be implicated by the mitochondrial apoptosis pathway after AgNPs/BER treatment. The results of this study agree with research indicating the important role of apoptotic biomarker genes Bax, Bcl-2, p53 in SCC cells [31]. *In vitro* studies have shown that AgNPs can induce and inhibit apoptosis by the enhanced expression of Bax and Bcl-2, respectively, suggesting that the OSCC cells’ fate depends on pro- and anti-apoptotic signals [27,32]. In conclusion, overexpression of Bcl-2 in SCC-25 treated by both agents AgNPs/BER and associated with a bidirectional attenuation effect, may play a role in the rational regulation of silver nanoparticles’ action. Additionally, this effect can protect normal cells from the cytotoxicity of silver nanoparticles and berberine.

### 2.4. Morphological Characteristic of SCC-25 Cells

**Figure 7:**
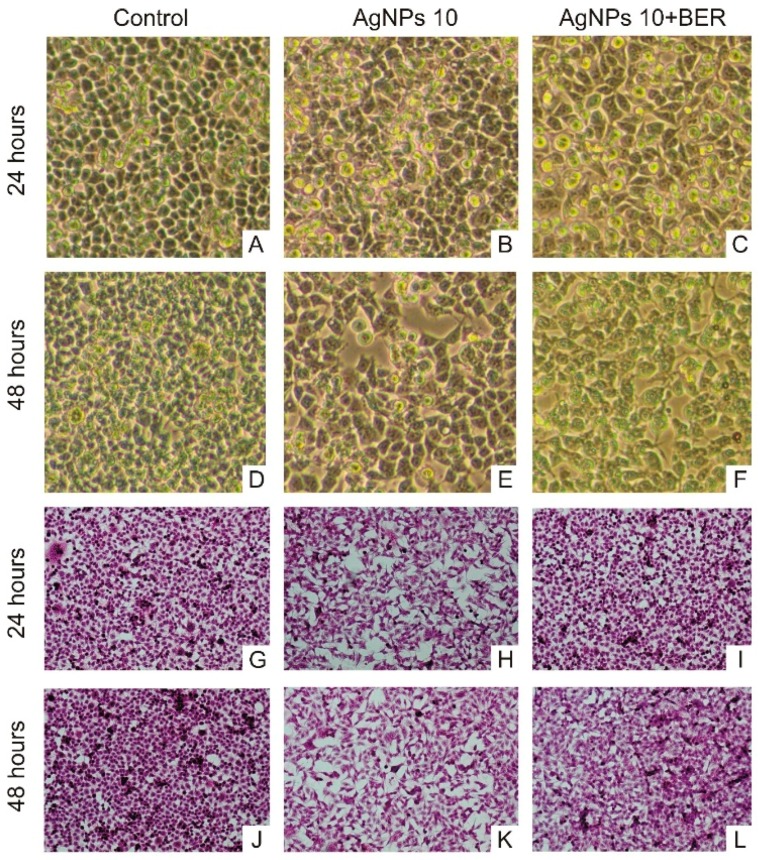
Microscopic images (magnification 10 × 10) of untreated SCC-25 cells and ones treated with 10 μg/mL AgNPs and 10 μg/mL AgNPs with 12.5 μg/mL BER (1/2 IC_50_ BER concentration). (**A**–**F**) Contrast-phase microscopy, (**G**–**L**) hematoxylin & eosin staining. Morphological changes of SCC-25 exposed to AgNPs include decreased numbers of cells, with less regular, non-round shapes, and morphology variations.

Microscopic characterization of squamous carcinoma cells (SCC) is shown in Figure 7. Following culture for 24 h and 48 h, AgNPs/BER-exposed SCC-25 cells demonstrated distinct morphological changes in optical micrographs (40×).

The results of our study strengthen the initial hypothesis that the application of silver nanoparticles might be potentially useful as a therapeutic medium or as an adjuvant for typical chemotherapeutics, potentiating their antiproliferative effects for oral cancer therapy. This study is one of the first to demonstrate that treatment of SCC-25 oral squamous cells cancer with low concentrations of AgNPs inhibits cell proliferation by induced cytotoxicity and dose-dependent cell death after longer incubation times. Moreover, we elucidated that addition of the natural alkaloid berberine exhibits an attenuating effect on the anti-proliferative activity of AgNPs, stimulating expression of pro-proliferative Bcl-2 gene and preserving the vitality of SCC-25 cancer cells. This non-synergistic phenomenon can be explained by aggregation of positively charged berberine with citrate-capped AgNPs dispersed in water solution, owing to the electrostatic repulsion between each other due to the negative charged surface [33].

A low energy orientation of the BER molecule with an optimal electrostatic state may induce interactions bridging the isoquinolinium positively charged nitrogen atom of BER and the negatively charged AgNPs. We assumed that the BER *vs.* AgNPs interaction was mainly aided by stabilization of the high negative charge density of AgNPs and positively charged BER with a “neutral” distribution of microstates to reach equilibrium. Under *in vitro* conditions the nanoparticles complexed with berberine showed lower cytotoxicity to tumor cells than the individual compounds alone. Considering the fact that both AgNPs and BER alone reveal a cytotoxic activity towards cancer cells, our results regarding the “antagonistic” effect of combination AgNPs + BER might have a practical implication considering an individually designed clinical protocol, with a bi-directional controlling effect of AgNPs *vs.* BER, and *vice versa*. The action may significantly reduce the potential side effects of silver nanoparticles or berberine applied alone. Considering the mode of BER/AgNP interactions, potentially higher concentrations of BER can impose a “saturation effect” with less attenuating influence of AgNPs towards BER. This shall be investigated in further studies.

Interestingly, BER alone was shown to be effective in inhibiting cell proliferation and promoting apoptosis in various cancerous cells. Recent studies suggest that berberine can suppress cell growth and reduce cell survival by arresting the cell-cycle [34]. It is believed that the binding interaction of berberine chloride with AgNPs in citrate buffer solution is directly linked with differently charged heterogenous molecules. The mode of binding of this alkaloid with AgNPs is debatable to date, with the potential modulation of the chemo-physical properties of BER in the presence of AgNPs. Nevertheless, based on our results, the cytotoxic effect of AgNPs in conjunction with BER can be inhibited, which indicates a non-specific antagonistic interaction between these two agents.

What is more, hypotethically, berberine may potently compete with AgNPs for binding sites within cell membranes and in that way may inhibit the anti-proliferative action of AgNPs alone. In conclusion, electrostatic activity with subsequent binding mode and BER molecule encapsulation, and also non-specific competition between BER and AgNPs are unambiguously at least two of the reasons for the reported attenuating effect of BER.

Our findings also suggest that low doses of AgNPs (up to 5 μg/mL) acting for a short period of time may not affect cancer cells’ viability and cell cycle. Here, we demonstrate that SCC-25 cells display variable susceptibilities to AgNPs under different sub-cytotoxic conditions, considering the incubation time. This is partially coherent with the recent results obtained by Jiao [35] which indicate that at low doses (2.0 mg/L), AgNPs may display a “hormesis effect” by accelerating cell proliferation and this effect is considered as an adaptive response of the carcinoma cells exposed to a “harmful environment”. However, the mechanisms and pathways by which colloidal silver induces cytotoxic activity on the SCC human cell line need further investigation. The biological effects of colloidal silver on cancer cells is still a current subject of scientific dispute. The induction of oxidative stress as a consequence of the generation of intracellular reactive oxygen species is the most commonly reported mechanism for AgNP toxicity [19,35]. Oxidative stress may elicit DNA damage and apoptosis [36]. Cancer cell death through the induction of apoptosis seems to be the crucial mechanism of AgNPs’ biological activity [36].

Initial studies have demonstrated that AgNP-treated human cancer cells (U251) exhibited chromosome instability and mitotic arrest under *in vitro* conditions [25]. A hypothetical mechanism of action of AgNPs links to the fact that the Ag^+^ ions which are released from the nanoparticles are believed to be involved in cell signaling cascades with the activation of Ca^2+^ release that further activates catabolic enzymes and damages mitochondrial membranes [25]. El Badawy *et al.* found that particle shape and size had a minimal influence on the toxicity of the evaluated AgNPs, while surface charge was a dominant factor in determining their toxicity [37]. Chang *et al*. demonstrated that the *in vitro* biological effect of silica-based nanoparticles depends significantly on the type and metabolic rate of the investigated cell line [38].

Results of the study carried out by Franco-Molina *et al.* [24] showed that MCF-7 breast cancer cells treated with colloidal silver displayed significantly reduced dehydrogenase activity, resulting in decreased NADH/NAD^+^, which in turn induces cell death due to decreased mitochondrial membrane potential. Gyenge *et al.* [7] provided evidence that certain types of nanoparticles can represent a useful adjuvant therapy for novel treatment protocols in human head and neck squamous cell carcinoma. Also, scanning electron microscopy analysis (SEM) of AgNP-treated human glioblastoma cells (U251) revealed their morphological changes, e.g., more spherical cells with minimal cellular extensions and low spreading patterns [25]. Cytological and morphological changes in carcinoma cells exposed to AgNPs may occur as a result of their interference with the structure and functions of the actin cytoskeleton [39]. Based on current knowledge [25], a long intracellular persistence of silver nanoparticles could be of advantage for long-term therapeutic modalities, including chemotherapy. As elucidated, the cytotoxic effect of silver nanoparticles towards either cancer or normal cells can be easily regulated by the controlling influence of berberine molecules. The potential long-term effects of AgNPs at low doses on humans should be further assessed to set a foundation for their rational application, particularly in case of oral cancers, taking into account the complexity of the interaction between AgNPs and oral soft tissues.

Due to the severe impact and prevalence of OSCC worldwide, this aspect could be of significant clinical relevance. Since the present data was gathered within an *in vitro* setting and a single cancer cell line, further investigations with focus on OSCC should take into account different nanoparticle parameters (type, size, agglomeration, solution stabilizer) and synergistic effects with various anti-cancer drugs to further support this potential intriguing aspect of cancer chemotherapy [40,41]. Additionally, further studies may reveal the cytotoxic and genotoxic effects of AgNPs towards endothelial progenitors, responsible for tumour angiogenesis [42]. These studies, including models of anti-neoplastic drug carrier-particles, may deliver novel findings providing insights on the suppression of human oral cancer. They may serve as useful tools for the development of novel treatment options in selected cases of OSCC.

## 3. Experimental Section

### 3.1. Colloidal Silver Nanoparticles and Berberine Characterization

Silver, dispersion nanoparticles (AgNPs), 10 nm particle diameter size (10 nm ± 4 nm TEM) were purchased from Sigma-Aldrich (St. Louis, MO, USA, catalog No. 730785). According to the product information, both of the AgNPs were supplied at a concentration of 0.02 mg/mL dispersed in aqueous buffer, containing 2 mM sodium citrate as a stabilizer to prevent aggregation (0.02 mg/mL ± 5% mass concentration). Correlations between nanoparticle diameter, mass concentration, and number concentration were as follow: nanoparticle diameter 10 nm = mass concentration 0.02 mg/mL = number concentration 3.6 × 10^12^ nanoparticles/mL. The rational selection of the AgNP concentration range in our experiments was supported by previous reports stating that a very low amount of silver is needed for their effective action and potential medical applications due to the negligible cytotoxic effect of AgNPs towards human cells at these concentration levels.

A dilute aqueous citrate buffer, which weakly associates with the nanoparticle surface, was used. This citrate-based and weakly bound capping agent provides long term stability and is readily displaced by various other molecules including thiols, amines, polymers, antibodies, and proteins [43]. Manufactured monodisperse silver nanoparticles were free from agglomeration, and extensively characterized for particle parameters by manufacturer using transmission electron microscopy (TEM) images, dynamic light scattering, Zeta potential measurements, and UV/Visible spectral analysis. The properties of the AgNPs used were the following: spherical particle morphology, cubic crystallographic structure, refractive index n_20_/D 1.333, density 0.997 g/mL at 25 °C, fluorescence λ_em_ 388 nm, FWHM 59 nm, wavelength 380–405 nm, absorbance 0.92–1.23 Abs UN [44].

**Figure 8:**
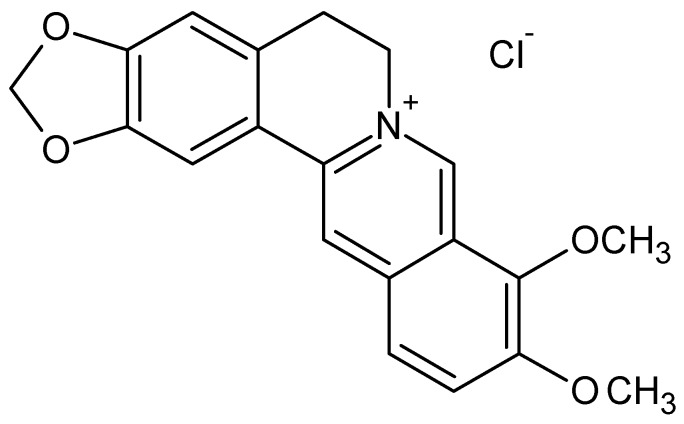
Chemical structure of the berberine derivative—berberine chloride salt, containing a protoberberine skeleton and nitrogen cation ion. The positively charged berberine molecule possesses a great affinity to react rapidly with negatively charged anions, e.g., silver nanoparticles. This mechanism depends on the electrostatic interaction between the charged surfaces and cationic berberine. Besides the electrostatic forces, biding interactions may also play a significant role in the attenuation effect of BER *vs.* AgNPs. Hypothetically, the BER molecules structure turns into a less biologically active form after encapsulation by AgNPs, which is followed by further AgNP aggregation. The alteration of BER to encapsulated form and the interplay of AgNPs surface charge determine the interaction between the alkaloid and AgNPs.

Berberine chloride (C_20_H_18_ClNO_4_, molecular weight 371.81, Figure 8) was purchased from Sigma Chemical Co. (St. Louis, MO, USA). Berberine, formulated as a chloride salt which eliminates solubility problems associated with the original plant extract compounds, was dissolved in deionized water and filtered through a 0.22 µm Millipore filter (Sartorius Co., Bohemia, NY, USA) before use.

### 3.2. Tongue Cancer Cells Culture

**Figure 9:**
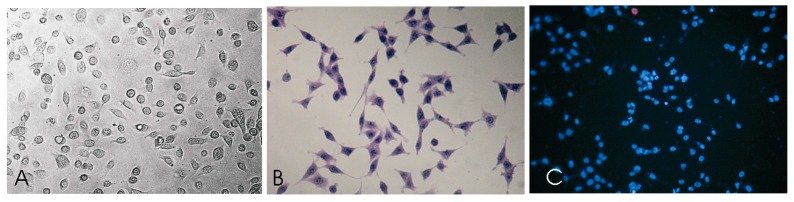
SCC-25 squamous cell carcinoma cell morphology and spreading pattern: nuclear enlargement, highly irregular shaped cells, dense chromatin, prominent nucleoli, cells are connected to each other through short and slender extensions (**A**) nonstained culture, untreated SCC-25 cells, optical magnification 400×). Cytological features of untreated SCC-25 carcinoma cells exhibiting characteristic signs of cellular atypia: nuclear and pleomorphic cytoplasmic pattern, increase nucleus-cytoplasm ratio, irregular nuclear shapes, hyperchromasia (**B**) hematoxylin & eosin staining, magnification 400×). Cells were costained (blue) to label DNA nucleic acids. Hoechst staining shows that most cells have round and intact nuclei (**C**) immunofluorescent staining of untreated cells using Hoechst 33342 fluorescent dye).

The SCC-25 human tongue squamous carcinoma cell line (catalogue no. ATCC CRL-1628, American Type Culture Collection, Manassas, VA, USA) was cultivated with the use of a 50:50 mixture of Dulbecco’s modified Eagle’s medium (DMEM) and Ham’s F12 medium with addition of 10% fetal bovine serum (FBS). Medium was supplemented with 400 ng/mL hydrocortisone and Antibiotic-Antimycotic Solution (GE Life Sciences/PAA Laboratories, Inc., Pittsburgh, PA, USA). The cancer cells were maintained and grown in monolayer cultures at 37 °C and 5% of carbon dioxide (CO_2_ incubator, Heraeus Instruments, Hanau, Germany). Subsequent passages were made by treating confluent cell culture with trypsin solution and then, cells were plated into a new cell culture receptacle in the ratio of 1:4. All reagents for cell culture were purchased from PAA Laboratories GmbH (GE Life Sciences/PAA Laboratories, Inc.) and Sigma-Aldrich Chemical Company. The cell culture was visually assessing with a Zeiss Axiostar binocular microscope (Carl Zeiss, Jena, Germany). Additionally, SCC-25 cells were stained in culture with the use of standard hematoxylin & eosin protocol and Hoechst 33342 dye for nuclear labeling. Figure 9 presents selected images of the investigated SCC-25 cells.

### 3.3. Cell Viability. 3-(4,5-Dimethylthiazol-2-yl)-5-(3-carboxymethoxyphenyl)-2-(4-sulfophenyl)-2H-tetrazolium (MTT) Assay

For experiment purpose, nanoparticles were ultrasonicated for 1 h directly prior to use in cell culture. The cytotoxicity of silver nanoparticles was measured by a commercial cell viability assay using the 3-(4,5-dimethyl-2-thiazyl)-2,5-diphenyl-2*H*-tetrazolium bromide (MTT) assay as previously described. In order to evaluate the AgNPs’ cytotoxic properties, cells of the examined line were plated on 96-well plates in the amount of 10,000 cells per well and then culture medium in the amount of 0.1 mL was added. Cells were left for 24 h in order to attach to the culture medium.

Taking the relatively low stock concentrations of the AgNPs into consideration (0.02 mg/mL, *i.e.*, 20 μg/mL), we chose five different concentrations of AgNPs for exposure in the subsequent experiments, starting from 1/2 of the initial stock concentration. After the lapse of this period, culture medium was decanted and to each well a culture medium containing AgNPs alone with concentrations 0.31, 0.62, 2.5, 5, 10 μg/mL and AgNPs at the highest concentration 10 μg/mL + BER (12.5 μg/mL) were added and left for 24 and 48 h. After the lapse of this period culture medium was decanted and to each well 10 μL of MTT reagent (Sigma-Aldrich) was added. Cells were left for 4 h After the lapse of this period, to each well 200 μL of DMSO (Sigma-Aldrich) was added, in order to dissolve formed formazan crystals. Controls included native cells and medium alone. The spectrophotometric absorbance at 570 nm was measured by microplate reader. In order to deduct the number of dead cells, the following formula was used: (cell death %) = [1-(absorbance of examined wells/absorbance of control wells)] × 100%.

### 3.4. Flow-Activated Flow Cytometry Analysis

Due to the fact that MTT assay revealed the attenuating effect of the combination of BER and AgNPs, flow cytometry analysis only examined the effect of AgNPs towards SCC-25. SCC-25 cells were seeded in 6-well plates and incubated with 10% medium at 37 °C (AgNPs concentrations: 0.31; 0.62; 2.5; 5; 10 μg/mL). Samples collected after 24 h and 48 h were gentle centrifuged (3 min at 1500 rpm, at RT) and washed in 1 × PBS, than centrifuged again. Obtained pellets were fixed in chilled 70% ethanol. Samples were kept in −20 °C until cell cycle assayed. After ethanol removal cells were suspended in 1 × PBS and warmed up to 37 °C. RNase (Sigma-Aldrich) digestion (100 µg/mL) were performed for next 20 min. Propidium iodide (Sigma-Aldrich), at a concentration of 100 µg/mL in PBS, was used for DNA labeling. After 15 min on ice the cell cycle was assayed by fluorescence-activated cell sorting analysis using a FACSAria III instrument (Becton Dickinson Biosciences, San Jose, CA, USA) with the following configuration: 488 nm laser line, LP mirror 566, BP filter 585/42.

### 3.5. BCL-2 and BAX Genes Expressions Using RT-QPRC Reaction

Based on the results of the MTT assay and flow cytometry, only one concentration of AgNPs—10 μg/mL—was used for further Bax and Bcl-2 genes expression assays, along with a fixed concentration of berberine represented as 1/2 IC_50_ BER (12.5 μg/mL). After completion of the incubation period for the culture of SCC-25 cells, supernatant was collected, and RNA was isolated in line with the procedure, by means of phenol-chloroform method of extraction, using the TRI Reagent kit (Zymo Research Corporation, Irvine, CA, USA), adding 1 mL of the reagent to each culture vessel. The efficiency of RNA isolation was assessed by spectrophotometry, as well as by means of agarose gel electrophoresis. Amplification was performed with the use of commercially available kits of primer and probe pairs (Applied Biosystems, Foster City, CA, USA) for each gene analyzed.

The quantitative PCR reaction, preceded by reverse transcription, was performed with the use of QuantiFast kit (Qiagen, Hilden, Germany). The kinetics of the reaction was determined by means of CFX Connect Real-Time System amplifier (Bio-Rad, Hercules, CA, USA), while the level of relative expression of studied genes (Bcl-2 and Bax) was calculated on the basis of the 2^−ddCt^ method. Specificity of the reaction of amplification of all fragments of studied genes was assessed by means of polyacrylamide gel electrophoresis. Each sample was examined in two repetitions, and for each the threshold amplification value (threshold cycle—Ct), that is the moment, in which the level of fluorescence exceeded the line that separates background fluorescence during analysis after RT-QPCR reaction. The analysis of results has been performed by means of comparative Ct method. In accordance with this method, ΔCt was calculated for each of the genes studied, using the formula: ΔCt of the studied gene = Ct of the studied gene − Ct of the reference gene.

Both in stimulated cells and in control, the GAPDH (glyceraldehyde-3-phosphate dehydrogenase) gene was assumed as reference gene, due to the fact that it is a gene whose expression remains at a stable level in all types of cells. The subsequent step concerned the calculation of ΔΔCt value, using the formula:

ΔΔCt of the studied gene = ΔCt of the studied gene in study material − ΔCt of the studied gene in control
(1)

For statistical analysis, the parameter was used, whose value amounted to 2^−ΔΔCt^. The calculation of standardized value of relative gene expression level in an unknown sample, in relation to control, is performed in accordance with the formula R = 2^−ΔΔCt^. The results thus obtained are expressed as multiplicity of the calibration sample. The value of parameter R equal to 1 means that the level of expression or number of gene copies in the calibration sample and the unknown sample is the same. A figure lower than one indicates the higher level of expression in the calibration sample, while a figure exceeding one indicates a higher expression in unknown sample, in comparison with the reference sample.

### 3.6. Statistical Analysis

The results are expressed as means ±SD obtained from three separate experiments performed in quadruplicates (*n* = 12) for cytotoxicity. The experimental means were compared to the means of untreated cells harvested in a parallel manner. Differences between 24 h-incubated samples and also for 48 h-incubated samples were tested for significance using the one- and multiple-way ANOVA Friedman ANOVA test (absorbance means values, different AgNPs concentrations). Differences between 24 h and 48 h of incubation time samples (absorbance means values) within the same AgNPs concentrations were tested for significance using subsequently a Wilcoxon test or *post-hoc* test (Statistica 9.0v, StatSoft, Tulsa, OK, USA). A *p*-value less than 0.05 were considered statistically significant.

## 4. Conclusions

Our study is one of the first to provide evidence that silver-based nanoparticles applied alone reduce the viability of human oral squamous cell carcinoma cells while the natural compound berberine diminishes their antiproliferative effect. However, their complex endocytotic pathways require thorough investigations. Further *ex vivo* and *in vivo* studies on oral malignant neoplasms should consider the combination of bioactive particles of certain types and sizes with specific chemotherapeutic drugs, representing a carrier driven anti-tumor drug delivery system.
